# Population history, gene flow, and bottlenecks in island populations of a secondary seed disperser, the southern grey shrike (*Lanius meridionalis koenigi*)

**DOI:** 10.1002/ece3.1334

**Published:** 2014-12-04

**Authors:** David P Padilla, Lewis G Spurgin, Eleanor A Fairfield, Juan Carlos Illera, David S Richardson

**Affiliations:** 1 School of Biological Sciences, University of East AngliaNorwich Research Park, Norwich, NR4 7TJ, U.K; 2 Island Ecology and Evolution Research Group, IPNA-CSICC/Astrofísico Francisco Sánchez 3, 38206 La Laguna, Tenerife, Canary Islands, Spain; 3 Research Unit of Biodiversity (UO-CSIC-PA), Oviedo UniversityCampus of Mieres, Research Building, 5th floor. C/Gonzalo Gutiérrez Quirós, s/n, 33600, Mieres, Asturias, Spain

**Keywords:** Canary Islands, diplochory, genetic structure, microsatellites, mtDNA, phylogeography

## Abstract

Studying the population history and demography of organisms with important ecological roles can aid understanding of evolutionary processes at the community level and inform conservation. We screened genetic variation (mtDNA and microsatellite) across the populations of the southern grey shrike (*Lanius meridionalis koenigi*) in the Canary Islands, where it is an endemic subspecies and an important secondary seed disperser. We show that the Canarian subspecies is polyphyletic with *L. meridionalis elegans* from North Africa and that shrikes have colonized the Canary Islands from North Africa multiple times. Substantial differences in genetic diversity exist across islands, which are most likely the product of a combination of historical colonization events and recent bottlenecks. The Eastern Canary Islands had the highest overall levels of genetic diversity and have probably been most recently and/or frequently colonized from Africa. Recent or ongoing bottlenecks were detected in three of the islands and are consistent with anecdotal evidence of population declines due to human disturbance. These findings are troubling given the shrike's key ecological role in the Canary Islands, and further research is needed to understand the community-level consequences of declines in shrike populations. Finally, we found moderate genetic differentiation among populations, which largely reflected the shrike's bottleneck history; however, a significant pattern of isolation-by-distance indicated that some gene flow occurs between islands. This study is a useful first step toward understanding how secondary seed dispersal operates over broad spatial scales.

## Introduction

For over a century evolutionary, biologists have studied island flora and fauna as a way of gaining insight into otherwise intractable ecological and evolutionary processes. Island archipelagos are simplified and replicated study systems that vary in their size, isolation, and ecology, thus providing “natural laboratories” for evolutionary research (MacArthur and Wilson 1967; Whittaker 1998). Moreover, due to their small population size and restricted range, many island species are of high conservation priority (IUCN 2004). Understanding ecological and evolutionary processes on island archipelagos is therefore important from both a pure and an applied perspective.

One important ecological process now well documented on (but not restricted to) islands is secondary seed dispersal, or diplochory, whereby a seed is eaten by a frugivorous species (e.g., a bird or lizard), which is in turn eaten and dispersed by a carnivorous species (e.g., a predatory bird). Numerous cases of secondary seed dispersal have been documented on islands (Nogales et al. 1998; Padilla et al. 2012), but little is known about the scale at which secondary seed-dispersing organisms structure plant communities. In many cases, predators disperse much longer distances than frugivorous species upon which they feed, and therefore, secondary seed dispersal is potentially important for structuring plant communities over regional scales (Higgins et al. 2003). Indeed, one potential as yet unexplored possibility is that secondary seed dispersers may be involved in moving seeds to and between oceanic islands. Study into the broad-scale distribution, population history, and ecology of secondary seed dispersers is an important first step toward a fuller understanding of how this process structures plant communities.

Genetic markers are powerful tools for making inferences about ecological and evolutionary processes that cannot be directly observed, including historical and recent changes in population size and patterns of migration (Avise 2004). The combined use of different markers with different properties is a particularly powerful approach, as it can help to disentangle the effects of evolutionary processes operating over different spatiotemporal scales (Parker et al. 1998; Zhang and Hewitt 2003). The use of molecular markers to study population history and gene flow among island populations has revealed complex and varied evolutionary processes across different groups (Juan et al. 2000; Emerson 2002). More studies across different taxonomic groups, particularly of species involved in key ecological processes, will further our understanding of evolutionary patterns and processes on islands.

Here, we use molecular markers to study population history, structure, and gene flow in an endemic subspecies of the southern grey shrike, *Lanius meridionalis koenigi,* in the Canary Islands. This predatory bird is found on eight islands and islets in the Canary Islands, where it inhabits open shrub habitats from the coast up to 2000 m above sea level (Martín and Lorenzo 2001). Recent molecular data suggest that the Canarian shrikes have a North African origin (Gonzalez et al. 2008; Klassert et al. 2008; Olsson et al. 2010), but their relationship between the Canarian and African subspecies has not yet been fully resolved. Within the Canary Islands, the southern grey shrike is an important secondary seed disperser, where it preys on frugivorous lizards and excretes viable seeds (Nogales et al. 1998; Padilla et al. 2012). Therefore, studying patterns of genetic diversity across the shrike populations could provide a foundation for new insight into broader ecological processes within and across the Canary Islands and highlight issues of conservation concern.

Our specific aims were as follows: we first determine the relationship between Canarian shrikes and subspecies from North Africa and estimate the degree of divergence between island and mainland populations. We then examine patterns of genetic structure between island populations and assess the roles of historical and recent bottlenecks and contemporary patterns of migration in structuring the island shrike populations. We then discuss the implications of our findings for the conservation and biology of the shrikes, and recommend future directions for further study into seed dispersal and habitat conservation within and across islands.

## Materials and Methods

### Study area and field sampling

**Figure 1:**
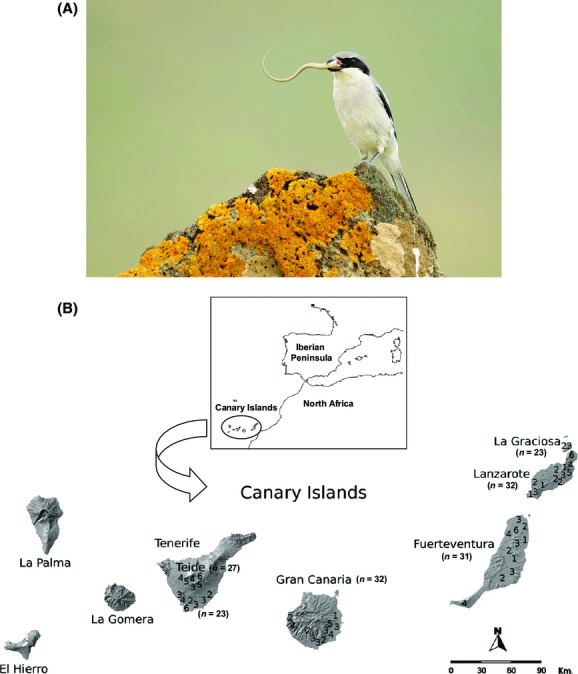
(A) Southern grey shrike (photo by José Juan Hernández); (B) Map of the Canary Islands showing the number (single digits) of southern grey shrikes caught per sampling site.

The Canarian subspecies of the southern grey shrike occupies four main islands in the Canarian archipelago (Tenerife, Gran Canaria, Fuerteventura, and Lanzarote), three islets off the north coast of Lanzarote (La Graciosa, Montaña Clara, and Alegranza), and one islet off the north of Fuerteventura (Lobos; Fig.1). Shrikes were captured from the populations of Gran Canaria, Fuerteventura, Lanzarote, and La Graciosa in April–June 2007, and on Tenerife from both the coastal population and the high mountain population on El Teide in December 2006, February 2007, and May and October 2008. The shrike populations on the small islets of Montaña Clara, Lobos, and Alegranza are very small – estimated at two, four, and 10 breeding pairs, respectively (Martín and Lorenzo 2001) – and were therefore not considered. Individuals were captured from a range of localities within each population to obtain a representative sample (Fig.1). All birds were captured using a modified potter trap, ringed with aluminum rings from the Spanish Ministry of Environment, and classified as juvenile or adult based on feather molt pattern. Blood samples (*c*. 40 *μ*L) were taken by brachial venipuncture and stored at room temperature in 1-mL screw-capped vials filled with 800 *μ*L absolute ethanol. All birds were released after sampling.

### Molecular methods

DNA was extracted using a standard salt method (Aljanabi and Martinez 1997). The quality and concentration of DNA was checked by visualization on a 2% agarose gel after electrophoresis, and samples were diluted to 10–50 ng/*μ*L. The sex of all individuals was determined using the molecular techniques described by Griffiths et al. (1998).

A 809-bp fragment of the mtDNA cytochrome *b* (cyt-*b*) gene was amplified in at least 11 individuals per population using the primers L-15035 and H-15985 (Klassert et al. 2008). PCRs consisted of 5 *μ*L TopTaq Master Mix (Qiagen, West Sussex, UK), 0.5 *μ*L (0.01 mmol/L) each primer, 2.5 *μ*L H_2_O, and 1.5 *μ*L DNA. Reaction conditions were as described by Klassert et al. (2008). Sequencing reactions, in both directions, consisted of 1 *μ*L PCR amplicon, 1 *μ*L BigDye terminator reaction mix, 0.15 *μ*L (0.01 mmol/L) of primers, 1.5 *μ*L sequencing buffer, and 6.35 *μ*L H_2_0. Sequencing reaction conditions were as follows: 94°C for 2 min, followed by 25 cycles of 96°C for 10 s, 50°C for 5 s, and 60°C for 4 min, with a final extension of 60°C for 1 min. Final PCR products were sequenced using an ABI Prism 3730 sequencer.

All individuals were genotyped at eight microsatellite loci identified as polymorphic in the southern grey shrike (Mundy and Woodruff 1996; Martinez et al. 1999; Dawson et al. 2010) (Appendix S1). The loci were amplified in three multiplex PCR reactions (Appendix S1) following Kenta et al. (2008). PCR conditions were as follows: 15 min at 95°C followed by 40 cycles of denaturation at 94°C for 30 s, annealing at 56°C for 90 s, and extension at 72°C for 60 s, with a final extra extension at 60°C for 30 min. PCR products were diluted 1 in 500 and run on an ABI 3730 DNA sequencer using ROX 500 size standard. Genotypes were scored in GeneMapper v. 3.7 (Applied BioSystems, Carlsbad, CA, USA).

### Analyses

Unless stated otherwise, all analyses were carried out using R version 2.14.1 (R Development Core Team 2008). Mitochondrial sequences were edited and aligned by eye in BioEdit 7.0.9 (Hall 1999). For each population, number of haplotypes, haplotype (h), and nucleotide diversities (*π*) with their standard deviations were calculated in DnaSP 5.0 (Rozas et al. 2003). To visualize the phylogenetic relationships among haplotypes, a statistical parsimony network of mitochondrial sequences was generated using TCS version 1.21 (Clement et al. 2000). In addition, all available cyt-*b* sequences of the southern grey shrike from GenBank were incorporated; these were from the Canary Islands (*L. m. koenigi*) and northern Africa (*L. m. elegans*,*L. m. algeriensis,* and *L. m. leucopygos*; Appendix S2). We also included sequences from African, European, and Asian populations of *L. m. uncinatus*,*L. somalicus*,*L. dorsalis*,*L. excubitor sibiricus,* and *L. e. homeyeri* (Olsson et al. 2010).

Prior to phylogenetic analyses, we estimated the model of evolution that best fits our mtDNA sequences using jModelTest version 0.1.1 (Posada 2008), and selected model HKY + I for all subsequent analyses. Phylogenetic relationships were assessed by Bayesian inference using MrBayes version 3.1.2 (Ronquist and Huelsenbeck 2003). Markov chains were run for 10 million generations and trees were sampled every 1,000 generations. The first 2,500 trees were discarded as burn-in generations. Two independent runs were performed in order to ensure that posterior probabilities were similar. Maximum likelihood analysis was also carried out using MEGA version 5.0.5 (Tamura et al. 2011) with branch supports evaluated using 10,000 bootstrap replicates. Both Bayesian and maximum likelihood trees were visualized using FigTree v. 1.3.1 (Rambaut 2009). We estimated divergence times of Canarian and North African shrike cyt-*b* haplotypes using BEAST 1.8.0 (Drummond and Rambaut 2007). We included sequences from *L. m. koenigi* and its closest relative, *L. m. elegans* (see Results). Analyses were carried out using constant size population priors, the Hasegawa-Kishino-Yano nucleotide substitution model and a strict clock method that assumed a rate of 2.1% sequence divergence per million years (Weir and Schluter 2008). We ran the program for 10 million generations, with a burn-in of 1 million generations, and then used Tracer v. 1.5 (Rambaut and Drummond 2009) to assess convergence and effective sample size of parameters and to generate the mean and 95% highest posterior density (HPD) estimates of divergence time.

At the microsatellites, Hardy–Weinberg (H-W) equilibrium for each locus and population, and linkage disequilibrium between all pairwise combinations of loci, were tested using GENEPOP (Raymond and Rousset 1995). The presence of null alleles was tested for using CERVUS v. 3.0.3 (Marshall et al. 1998). For each locus and population, allelic richness and heterozygosity were calculated using Arlequin version 3.5 (Excoffier and Lischer 2010), and differences between islands were tested using mixed models, with island identity as a fixed factor and locus identity as a random factor, implemented in the nlme package in R (Pinheiro et al. 2013). We used two approaches to test for genetic bottlenecks at the microsatellite loci – for each population, we calculated *M,* a ratio based on the number of alleles to the allelic size range (Garza and Williamson 2001) in Arlequin, and ran a Wilcoxon test for heterozygosity excess (Cornuet and Luikart 1996) using the program BOTTLENECK (Piry et al. 1999). For the latter, we used a two-phase mutation model which we ran twice for each population, assuming that the percentage of stepwise mutations was 90% and 80%, respectively.

To visualize overall genetic structure among the Canarian shrike populations, we carried out a principal component analysis of the eight microsatellite loci, using the Adegenet package in R (Jombart 2008). We also carried out a Bayesian analysis of genetic structure in the program STRUCTURE v. 2.1 (Pritchard et al. 2000). We used an admixture model, allowing the number of genetic clusters (*K*) to vary between 1 and 6. For each run, a burn-in of 10,000 steps with 500,000 MCMC repetitions was used. A total of four independent runs were completed for each value of *K*, and we compared average log probability of data for each value of *K* to determine the most likely number of genetic clusters. In addition, we calculated the statistic Δ*K*, which is based on the rate of change in the log probability of data between successive *K* values (Evanno et al. 2005). Pairwise *F*_ST_ values for both microsatellite and mtDNA were calculated in Arlequin. Pairwise values of *D*_EST_ (Jost 2008) were calculated using the program SMOGD (Crawford 2010). Correlations between matrices of genetic structure, as well as correlations between genetic and geographic distance (measured as the closest straight-line distance between islands), were tested using Mantel tests implemented in the Ecodist package in R (Goslee and Urban 2007).

## Results

**Figure 2:**
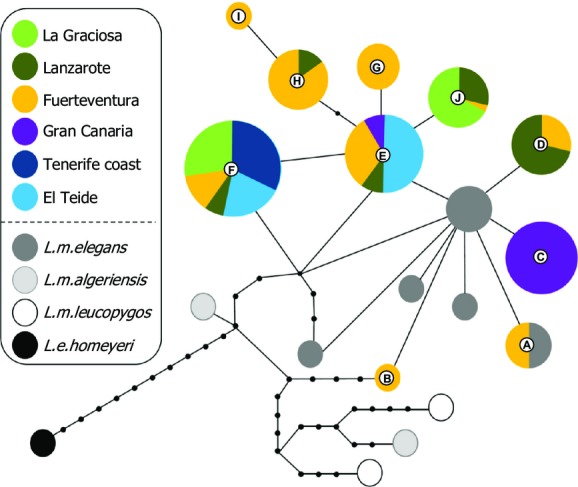
Minimum spanning network based on mtDNA sequences (cytochrome *b*) showing relationships among the ten different haplotypes of Canary Island shrikes (colored) and how these are connected to haplotypes from shrikes from northern Africa (gray, white and black). Circle sizes are proportional to haplotype frequency. Lines represent one mutational change at one nucleotide site, and black dots represent missing haplotypes.

A total of 106 individuals were sequenced for cyt-*b* (Table1), from which 10 different haplotypes were detected. Shrikes from Fuerteventura shared one haplotype with *L. m. elegans* (Fig.2, haplotype A), while another four haplotypes were directly connected (only one base pair different) to other haplotypes of shrikes from northern Africa (Fig.2B–E). Within the Canarian populations, two haplotypes were widespread, shared by individuals from most of the island populations (Fig.2E and F). The highest haplotype and nucleotide diversity in the archipelago was found in the eastern islands (Fuerteventura, Lanzarote and La Graciosa; Table1) with seven derived haplotypes present in these populations (Fig.2A,B,D,G–J), of which three belonged exclusively to Fuerteventura (Fig.2B,G, and I). Gran Canaria had the lowest haplotype and nucleotide diversity, and on this island, the most common haplotype (found in 15 of 17 of individuals) was unique to the island (Table1, Fig.2C). Pairwise *F*_ST_ distances among all Canarian populations based on mtDNA were high and significant, with the highest levels of structure between pairwise comparisons involving Gran Canaria, Tenerife coast, and Tenerife Teide (Table2).

**Table 1:** Mitochondrial DNA diversity (cytochrome *b*) of the southern grey shrike in the Canary Islands

Population	*n*	Number of haplotypes (h)	Haplotype diversity (Hd ± SD)	Nucleotide diversity (*π *± SD)
Tenerife Teide	15	2	0.476 ± 0.092	0.0006 ± 3.8^−4^
Tenerife coast	11	1	–	–
Gran Canaria	17	2	0.233 ± 0.126	0.0001 ± 5.2^−4^
Fuerteventura	26	9	0.833 ± 0.056	0.0027 ± 9.7^−4^
Lanzarote	16	5	0.714 ± 0.081	0.0024 ± 9.2^−4^
La Graciosa	21	2	0.526 ± 0.040	0.0013 ± 4.9^−4^
Total	106	8	0.815 ± 0.018	0.0022 ± 7.2^−4^

**Table 2:** Pairwise *F*_ST_ values estimated with mtDNA sequences (below the diagonal) and microsatellite data (above the diagonal)

*F*_ST_	TEID	TF	GC	FV	LZ	GRAC
TEID	–	0.067	0.205	0.221	0.129	0.271
TF	0.603	–	0.179	0.201	0.119	0.195
GC	0.782	0.903	–	0.098	0.082	0.108
FV	0.096	0.352	0.583	–	0.080	0.057
LZ	0.262	0.521	0.526	0.098	–	0.056
GRAC	0.233	0.417	0.721	0.225	0.224	–

TEID, Tenerife Teide; TF, Tenerife coast; GC, Gran Canaria; FV, Fuerteventura; LZ, Lanzarote; GRAC, La Graciosa.

All pairwise comparisons were statistically significant (*P *<* *0.05).

Both the Bayesian inference and maximum likelihood tree showed an identical, well-supported topology (Appendix S3). Phylogenetic analyses grouped the Canary Island shrikes with the North African subspecies with high nodal support. *Lanius m*.* elegans* from Tunisia and Mauritania were polyphyletic with shrikes from the Canaries, while shrikes from Algeria, Tunisia, and Chad (*L. m. algeriensis* and *L*.* m. leucopygos*) were grouped with low nodal support as a terminal lineage within this clade (Appendix S3). Estimates of divergence time suggested that cyt-*b* sequences from *L. m. koenigi* and *L. m. elegans* shared a single common ancestor 284,000 years ago (95% HPD estimates = 89,000–381,000 years ago).

**Figure 3:**
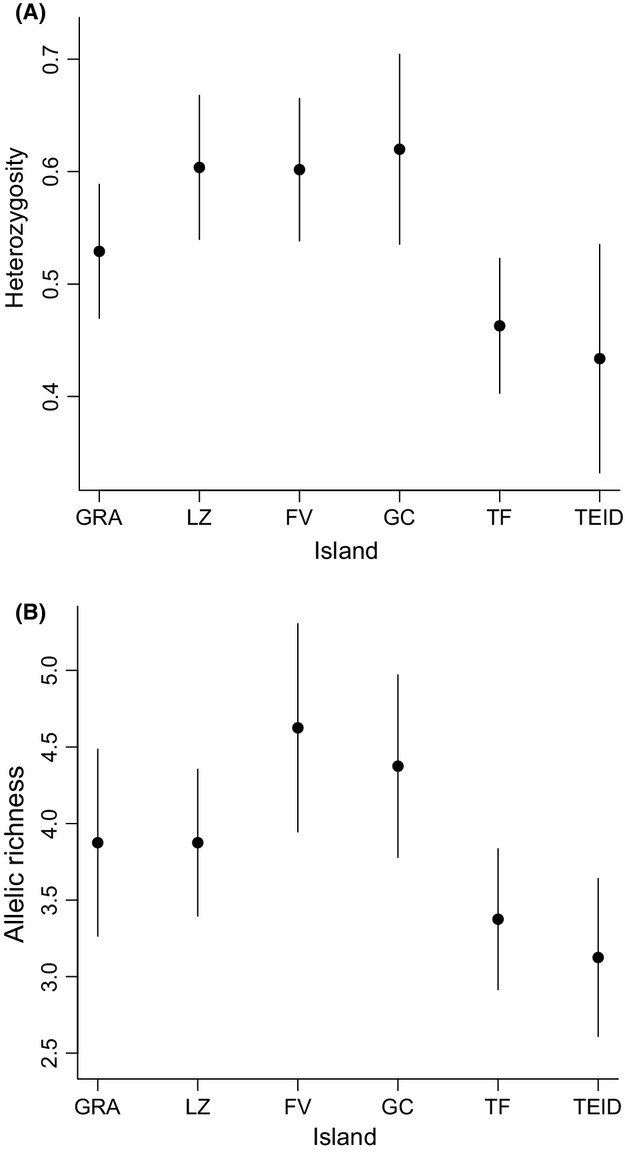
Average (mean ± SE) (A) heterozygosity and (B) allelic richness at eight microsatellite loci in Canarian shrike populations (GRA = La Graciosa, LZ = Lanzarote, FV = Fuerteventura, GC = Gran Canaria, TF = Tenerife coast, TEID = Tenerife Teide). Sample sizes for each population are given in Table3.

A total of 166 individuals were genotyped at the microsatellite loci (see Table3 for sample sizes per population). No loci were found to be in H–W disequilibrium in more than one population (all *P > *0.05), and no loci were found to be in linkage disequilibrium. Null allele frequencies were <0.1 at all eight loci. Differences in genetic diversity across populations were marginally nonsignificant when heterozygosity was considered (*F*_5,35_* *= 2.32, *P *=* *0.06) and marginally significant for allelic richness (*F*_5,35_* *= 2.58, *P *=* *0.04). Heterozygosity was highest in Fuerteventura, Gran Canaria, and Lanzarote (Fig.3A), and allelic richness was highest in Fuerteventura (Fig.3B). Both measures of genetic diversity were lowest in Tenerife coast and Tenerife Teide (Fig.3). Tests for genetic bottlenecks yielded mixed results. The *M* ratio of allelic richness to allelic size range (Garza and Williamson 2001) was lowest in Tenerife coast and Tenerife Teide and indicative of a bottleneck in these populations, whereas tests for heterozygosity excess suggested that the islands of Gran Canaria and Lanzarote have experienced recent bottlenecks (Table3). The latter test was robust to changes in the percentage of stepwise mutations, with similar results at both 80% and 90% (Table3).

**Table 3:** Results of tests for genetic bottlenecks in Canarian shrike populations, using microsatellite loci (see Fig.1 for sample sizes): *M* (Garza and Williamson 2001) and Wilcoxon tests for heterozygote excess (Piry et al. 1999). The Wilcoxon tests were carried out twice, assuming that the percentage of stepwise mutations (PSM) was 90 and 80, respectively. Values highlighted in bold are those indicative of a bottleneck (*M *<* *0.68 for the Garza–William ratio *and P ≤ *0.05 for the Wilcoxon tests)

Population	*n*	*M*	*P*_(Hz excess, PSM = 90)_	*P*_(Hz excess, PSM = 80)_
Tenerife Teide	26	**0.55**	0.23	0.19
Tenerife coast	23	**0.62**	0.52	0.37
Gran Canaria	30	0.77	**0.03**	**0.01**
Fuerteventura	31	0.82	0.37	0.32
Lanzarote	32	0.71	**0.02**	**0.01**
La Graciosa	22	0.70	0.68	0.47

**Figure 4:**
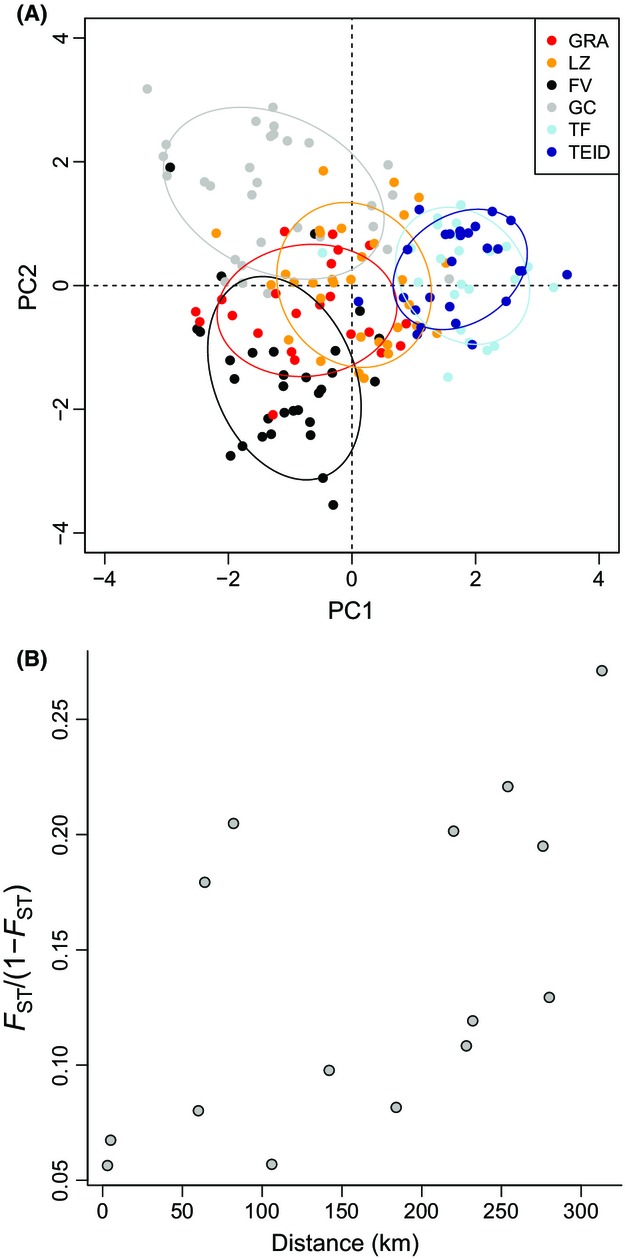
Genetic structure in Canarian shrike populations. (A) Principal component analysis of all loci implemented in the Adegenet package in R (PC1 and PC2 plotted on *x* and *y* axes, respectively). Ellipses represent 95% confidence intervals. (B) Pairwise (microsatellite) genetic distance in relation to geographic distance between populations.

A PCA of the microsatellite loci indicated moderate differentiation among populations, with birds from Tenerife coast, Tenerife Teide, and Gran Canaria most divergent from the other populations (Fig.4A). Bayesian analyses in STRUCTURE yielded similar results: depending on the method used, STRUCTURE suggested either two (highest mean Ln probability of data) or four (highest Δ*K*) clusters, both of which corresponded roughly to geographic distribution (Appendix S4). Assuming *K *=* *2, one cluster represented individuals from Tenerife (both Teide and coast), one from the other populations, whereas assuming *K *=* *4 yielded the Tenerife cluster, plus one cluster corresponding to Gran Canaria, one to Fuerteventura, and one to Lanzarote and La Graciosa combined (Appendix S4). As with the PCA, STRUCTURE did not separate the shrike populations into clear genetic groups, instead suggesting either admixture or a lack of resolution from the microsatellite loci (Appendix S4).

Pairwise values of *F*_ST_ and *D*_EST_ at the microsatellite loci were highly correlated (Mantel test: *R *=* *0.85, *P < *0.001), and thus, only *F*_ST_ was used in subsequent analyses. All pairwise comparisons of differentiation based on microsatellite markers were significant, with highest levels of structure found between Tenerife and the other populations (Table2). A positive correlation was observed between geographic and genetic distance matrices, suggesting an overall pattern of isolation-by-distance (*R *=* *0.58, *P *=* *0.03; Fig.4B) – however, the isolation-by-distance plot showed two distinct groups along the *y*-axis, with comparisons involving Tenerife coast and Tenerife Teide not fitting into the overall isolation-by-distance pattern (top five points on Fig.4B; Table2). We therefore carried out a partial Mantel test for isolation-by-distance, controlling for whether Tenerife coast or Tenerife Teide was involved in pairwise comparisons, the result of which was marginally nonsignificant (*R *=* *0.51, *P *=* *0.08).

## Discussion

The Canary Islands have proved excellent models for studying biogeography, especially with regard to colonization, dispersal, and population history (Juan et al. 2000; Illera et al. 2012; Spurgin et al. 2014). This study set out to assess patterns of genetic variation in the southern grey shrikes, currently classified as an endemic subspecies to this archipelago, both in relation to mainland populations and across islands. We found that the Canarian shrike populations are characterized by recent colonization events from the mainland, by historical and recent bottlenecks, and by a limited amount of interisland dispersal.

Our phylogenetic results are in accordance with the previous findings that *L. m. koenigi* is most closely related to the North African subspecies *L. m. algeriensis* and *L. m. elegans*, suggesting a North African origin (Gonzalez et al. 2008; Klassert et al. 2008; Olsson et al. 2010). However, we show for the first time that *L. m. koenigi* is not a monophyletic group and instead shares haplotypes with North African subspecies. While other Canarian species also have a North African origin (Illera et al. 2007), few are as closely related to African populations as we have observed here. The polyphyly of Canarian and North African shrikes, and the short branch lengths on the mtDNA phylogeny, suggest that multiple, recent colonizations have occurred from Africa to the Canary Islands. The number of haplotypes endemic to Fuerteventura, the high haplotype diversity and the fact that it shares haplotypes with continental populations suggests that this island has been most recently and/or frequently colonized from Africa. The highest levels of overall microsatellite variation are also found on Fuerteventura (Fig.3), and colonization from Africa via this island makes geographical sense as it is the closest to the mainland. We suggest that due to the genetic similarity and polyphyly, Canary Island shrikes should be, at least based on the genetic evidence, considered as populations of the *L. m. elegans* subspecies.

We found pronounced differences in genetic variation among populations, which are most likely the product of a combination of historical and recent colonization events, bottlenecks, and gene flow. While the general pattern of higher genetic variation in the Eastern islands held true for both mtDNA and microsatellite markers, subtle differences in variation across marker types exist (Table1, Fig.2). Most notable in this respect is Gran Canaria, which has relatively high microsatellite diversity but low mtDNA diversity and an exclusive mtDNA haplotype at high frequency, possibly reflecting a historical bottleneck. Interestingly, exclusively allopatric lineages in Gran Canaria have been found in other avian species, such as robins (*Erithacus rubecula*), blue tits (*Cyanistes teneriffae*), and common chaffinches (*Fringilla coelebs*) (Illera et al. 2012). The reason for these interesting patterns is as yet unclear, but possible explanations could be disease, patterns of volcanism, or past human disturbance (Illera et al. 2012).

Specific tests for recent bottlenecks using microsatellites yielded surprising results, with *M* ratios suggesting a bottleneck in Tenerife coast and Tenerife Teide, while tests for heterozygosity excess suggested bottlenecks in Gran Canaria and Lanzarote (Table3). Interestingly, this fits with anecdotal reports of shrike population declines on these islands due to human disturbance (Martín and Lorenzo 2001). Detection of a bottleneck using *M* ratios but not heterozygosity excess is expected when a bottleneck is older, more severe, and/or the population has recovered, whereas a signal of heterozygosity excess but not reduced *M* ratios is expected when the bottleneck is weaker and more recent (Williamson-Natesan 2005). It is therefore possible that Canarian shrikes have at some point in the more distant past undergone a severe, prolonged bottleneck in Tenerife and have experienced more recent, population declines in Gran Canaria and Lanzarote. These recent declines may be due to human disturbance and are troubling as the shrikes play an important role as seed dispersers on these islands (Padilla et al. 2012), and the broader ecological consequences of a decline in shrike populations is unknown. Further population monitoring and modeling is now required in order to assess whether and where conservation efforts should be targeted.

There are increasingly complex and efficient approaches to directly estimate patterns of migration and test hypotheses of colonization history from genetic data (e.g., Beerli & Felsenstein, 2001; Cornuet et al., 2008). However, these approaches either make assumptions about migration–drift equilibrium, or require a clearly definable set of alternative colonization scenarios and prior information about effective population sizes, past demography, and colonization times (Beerli, 2009; Beaumont, 2010). The shrike populations violate the assumption of migration–drift equilibrium, and we have little prior information with which to inform analyses. When combined with a limited number of markers, as we have here, violation of these assumptions may lead to false or misleading inferences. For this reason, we have not included analyses of directional gene flow, or approximate Bayesian computation (ABC) estimates of colonization history. Yet even with our descriptive approach, it is clear that bottlenecks and colonization history have most likely been the main drivers of genetic structure among the Canarian shrike populations. Moreover, we are able to make some tentative inferences about migration between islands based on our results. We observed a pattern of isolation-by-distance between most islands, but with elevated structure in pairwise comparisons involving the bottlenecked populations of Tenerife coast and Tenerife Teide (Table2, Fig.4B). Similar patterns have been observed in other studies (e.g., Worley et al. 2004) and in this instance indicate migration between neighboring populations, but limited dispersal between more geographically separated islands, and between Tenerife and the other populations.

Given the patterns of structure observed here, and the evidence for multiple colonization from the African mainland, it is clear that there is potential for shrikes to act as secondary seed dispersers both from Africa to the Canary Islands, and among the Canary Islands. It certainly seems likely that shrikes play a greater role in interisland seed dispersal than primary seed dispersers such as lizards or frugivorous bird species, which tend to be more differentiated from the mainland and exhibit less gene flow among islands (Suárez et al. 2009; Cox et al. 2010). A comparative phylogeographic approach, combining DNA sequence data from shrike and plant species, is likely to be the most rewarding approach in refining the extent to which this species structures plant communities across island populations.

## Data Archiving

Sequence data have been submitted to GenBank: accession numbers KC175566–KC175572.

## Supporting Information

**Appendix S1.**Eight microsatellite loci amplified in southern grey shrike (*Lanius meridionalis koenigi*) populations from the Canary Islands (references in bibliography of main text). **Appendix S2.** Cytochrome *b* sequences used in the present study. **Appendix S3.** Bayesian consensus tree based on mtDNA cytochrome *b*. Numbers are Bayesian posterior probabilities/bootstrap values of the maximum likelihood. **Appendix S4.** Output from the Bayesian clustering algorithm implemented in the STRUCTURE software, summarizing genetic structure at microsatellite loci across Canarian shrike populations.
