# Supplementary material for: Population history, gene flow, and bottlenecks in island populations of a secondary seed disperser, the southern grey shrike (Lanius meridionalis koenigi)
Source: Ecol Evol. 2014 Dec 4;5(1):36–45. doi: 10.1002/ece3.1334 (PMC4298432; doi:10.1002/ece3.1334)
Supplement: Supplementary file 1 — Appendix S1.Eight microsatellite loci amplified in southern grey shrike (Lanius meridionalis koenigi) populations from the Canary Islands (references in bibliography of main text). Appendix S2. Cytochrome b sequences used in the present study. Appendix S3. Bayesian consensus tree based on mtDNA cytochrome b. Numbers are Bayesian posterior probabilities/bootstrap values of the maximum likelihood. Appendix S4. Output from the Bayesian clustering algorithm implemented in the STRUCTURE software, summarizing genetic structure at microsatellite loci across Canarian shrike populations. [file ece30005-0036-sd1.docx]

**Appendix 1.** Eight microsatellite loci amplified in southern grey shrike (*Lanius meridionalis koenigi*) populations from the Canary Islands (references in bibliography of main text).

| **Multiplex** | **Locus** | **Reference** |
| --- | --- | --- |
| 1 | LS1 | (Mundy & Woodruff 1996) |
|  | LS2 | (Mundy & Woodruff 1996) |
|  | PPI2 | (Martínez *et al*. 1999) |
| 2 | LS3 | (Mundy & Woodruff 1996) |
|  | LTMR7 | (McDonald & Potts unpublished) |
|  | TG04-041 | (Dawson *et al*. 2010) |
|  | TG05-053 | (Dawson *et al*. 2010) |
| 3 | TG13-009 | (Dawson *et al*. 2010) |

**Appendix 2.** Cytochrome *b* sequences used in the present study. TEID: Teide. TF: Tenerife coast. GC: Gran Canaria. FV: Fuerteventura. LZ: Lanzarote. GRAC: La Graciosa.

| **Species/Subspecies** | **Locality**  **in GenBank** | **Localities (number of individuals**  **With the haplotype in the present study)** | **GenBank Accession** | **References** |
| --- | --- | --- | --- | --- |
| *Lanius meridionalis koenigi* | Tenerife | TEID(5), TF(11), FV(4), LZ(1), GRAC(10) | EF635029 | González et al (2008) |
|  | Lanzarote | TEID(10), GC(2), FV(6), LZ(2) | EF635013 | González et al (2008) |
|  | Gran Canaria | GC(15) | EF635028 | González et al (2008) |
|  | Fuerteventura | FV(3), LZ(6) | GU253515 | Olsson et al. (2010) |
|  | Fuerteventura | FV(6), LZ(1) | GU253511 | Olsson et al. (2010) |
|  | Fuerteventura | FV(1) | EF635027 | González et al (2008) |
|  | Fuerteventura | FV(1) | KC175566 | Present study |
|  | Fuerteventura | FV(3) | KC175567 | Present study |
|  | Lanzarote | FV(1), LZ(6), GRAC(11) | KC175568 | Present study |
|  | Fuerteventura |  | EF635026 | González et al (2008) |
|  | Tenerife |  | EF635030 | González et al (2008) |
|  | Fuerteventura |  | GU253512 | Olsson et al. (2010) |
|  | Fuerteventura |  | GU253513 | Olsson et al. (2010) |
|  | Fuerteventura |  | GU253514 | Olsson et al. (2010) |
|  | Fuerteventura |  | GU253516 | Olsson et al. (2010) |
|  | Fuerteventura |  | GU253517 | Olsson et al. (2010) |
|  | Fuerteventura |  | GU253518 | Olsson et al. (2010) |
| *Lanius meridionalis elegans* | Mauritania |  | GU253492 | Olsson et al. (2010) |
|  | Mauritania |  | GU253493 | Olsson et al. (2010) |
|  | Mauritania |  | GU253494 | Olsson et al. (2010) |
|  | Mauritania |  | GU253495 | Olsson et al. (2010) |
|  | Tunisia | FV(1) | GU253496 | Olsson et al. (2010) |
|  | Tunisia |  | GU253497 | Olsson et al. (2010) |
| *Lanius meridionalis algeriensis* | Algeria |  | GU253478 | Olsson et al. (2010) |
|  | Algeria |  | GU253479 | Olsson et al. (2010) |
|  | Tunisia |  | GU253477 | Olsson et al. (2010) |
| *Lanius meridionalis leucopygos* | Chad |  | GU253522 | Olsson et al. (2010) |
|  | Chad |  | GU253523 | Olsson et al. (2010) |
| *Lanius meridionalis uncinatus* | Socotra |  | GU253546 | Olsson et al. (2010) |
| *Lanius somalicus* | Chad |  | GU253562 | Olsson et al. (2010) |
| *Lanius dorsalis* | Kenya |  | GU253474 | Olsson et al. (2010) |
| *Lanius excubitor sibiricus* | Russia |  | GU253545 | Olsson et al. (2010) |
| *Lanius excubitor homeyeri* | Russia |  | GU253509 | Olsson et al. (2010) |

**Appendix 3.** Bayesian consensus tree based on mtDNA cytochrome *b*. Numbers are Bayesian posterior probabilities / bootstrap values of the maximum likelihood. TEID: Teide. TF: Tenerife coast. GC: Gran Canaria. FV: Fuerteventura. LZ: Lanzarote. GRAC: La Graciosa

0.4

*Lanius*

*meridionalis*

*koenigi*

LZ

*Lanius*

*meridionalis*

*elegans*

*Lanius*

*meridionalis*

*koenigi*

FV

*Lanius*

*meridionalis*

*koenigi*

TEID

*Lanius*

*meridionalis*

*koenigi*

FV

*Lanius*

*meridionalis*

*koenigi*

GC

*Lanius*

*meridionalis*

*koenigi*

GC

*Lanius*

*meridionalis*

*unciatus*

*Lanius*

*meridionalis*

*elegans*

*Lanius*

*meridionalis*

*algeriensis*

*Lanius*

*meridionalis*

*koenigi*

FV

*Lanius*

*meridionalis*

*koenigi*

FV

*Lanius*

*meridionalis*

*koenigi*

FV

*Lanius*

*meridionalis*

*koenigi*

LZ

*Lanius*

*excubitor*

*sibiricus*

*Lanius*

*meridionalis*

*koenigi*

TF

*Lanius*

*dorsalis*

*Lanius*

*meridionalis*

*algeriensis*

*Lanius*

*meridionalis*

*koenigi*

GRAC

*Lanius*

*meridionalis*

*koenigi*

TEID

*Lanius*

*meridionalis*

*koenigi*

GRAC

*Lanius*

*meridionalis*

*koenigi*

FV

*Lanius*

*meridionalis*

*koenigi*

LZ

*Lanius*

*meridionalis*

*leucopygos*

*Lanius*

*meridionalis*

*koenigi*

LZ

*Lanius*

*meridionalis*

*leucopygos*

*Lanius*

*meridionalis*

*elegans*

*Lanius*

*meridionalis*

*koenigi*

LZ

*Lanius*

*excubitor*

*homeyeri*

*Lanius*

*meridionalis*

*koenigi*

FV

*Lanius*

*somalicus*

*Lanius*

*meridionalis*

*koenigi*

LZ

*Lanius*

*meridionalis*

*koenigi*

FV

0.76 / 56

1.0 / 87

0.96 / 92

1.0 / 97

0.9 / 63

0.8 / 63

0.77 / 66

0.61 / 41

1.0 / 94

0.59 / 43

1.0 / 86

0.99 / 63

*Lanius*

*meridionalis*

*elegans*

*Lanius*

*meridionalis*

*elegans*

*Lanius*

*meridionalis*

*koenigi*

FV

*Lanius*

*meridionalis*

*koenigi*

FV

0.99 / 69

0.4

*Lanius*

*meridionalis*

*koenigi*

LZ

*Lanius*

*meridionalis*

*elegans*

*Lanius*

*meridionalis*

*koenigi*

FV

*Lanius*

*meridionalis*

*koenigi*

TEID

*Lanius*

*meridionalis*

*koenigi*

FV

*Lanius*

*meridionalis*

*koenigi*

GC

*Lanius*

*meridionalis*

*koenigi*

GC

*Lanius*

*meridionalis*

*unciatus*

*Lanius*

*meridionalis*

*elegans*

*Lanius*

*meridionalis*

*algeriensis*

*Lanius*

*meridionalis*

*koenigi*

FV

*Lanius*

*meridionalis*

*koenigi*

FV

*Lanius*

*meridionalis*

*koenigi*

FV

*Lanius*

*meridionalis*

*koenigi*

LZ

*Lanius*

*excubitor*

*sibiricus*

*Lanius*

*meridionalis*

*koenigi*

TF

*Lanius*

*dorsalis*

*Lanius*

*meridionalis*

*algeriensis*

*Lanius*

*meridionalis*

*koenigi*

GRAC

*Lanius*

*meridionalis*

*koenigi*

TEID

*Lanius*

*meridionalis*

*koenigi*

GRAC

*Lanius*

*meridionalis*

*koenigi*

FV

*Lanius*

*meridionalis*

*koenigi*

LZ

*Lanius*

*meridionalis*

*leucopygos*

*Lanius*

*meridionalis*

*koenigi*

LZ

*Lanius*

*meridionalis*

*leucopygos*

*Lanius*

*meridionalis*

*elegans*

*Lanius*

*meridionalis*

*koenigi*

LZ

*Lanius*

*excubitor*

*homeyeri*

*Lanius*

*meridionalis*

*koenigi*

FV

*Lanius*

*somalicus*

*Lanius*

*meridionalis*

*koenigi*

LZ

*Lanius*

*meridionalis*

*koenigi*

FV

0.76 / 56

1.0 / 87

0.96 / 92

1.0 / 97

0.9 / 63

0.8 / 63

0.77 / 66

0.61 / 41

1.0 / 94

0.59 / 43

1.0 / 86

0.99 / 63

*Lanius*

*meridionalis*

*elegans*

*Lanius*

*meridionalis*

*elegans*

*Lanius*

*meridionalis*

*koenigi*

FV

*Lanius*

*meridionalis*

*koenigi*

FV

0.99 / 69

**Appendix 4.** Output from the Bayesian clustering algorithm implemented in the STRUCTURE software, summarising genetic structure at microsatellite loci across Canarian shrike populations. Each vertical line represents an individual, and colours represent the proportion of genetic variation assigned to each genetic cluster (*K* – see text for details). Data were visualised assuming **A)** *K* = 2, and **B)** *K* = 4 – the two most supported values (see text for details).

**A**


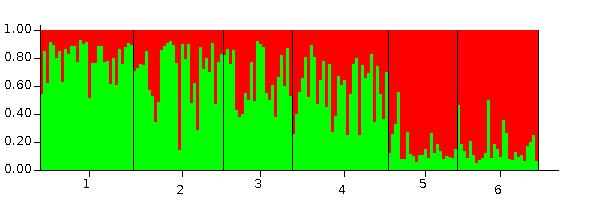


**B**


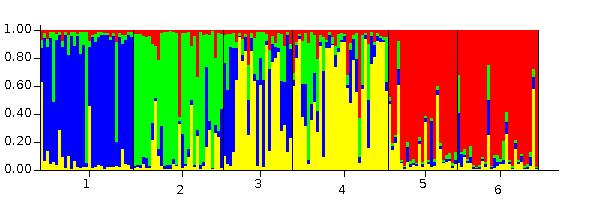


Gran Canaria Fuerteventura La Graciosa Lanzarote Tenerife El Teide
